# Selective inhibitor of Wnt/β-catenin/CBP signaling ameliorates hepatitis C virus-induced liver fibrosis in mouse model

**DOI:** 10.1038/s41598-017-00282-w

**Published:** 2017-03-23

**Authors:** Yuko Tokunaga, Yosuke Osawa, Takahiro Ohtsuki, Yukiko Hayashi, Kenzaburo Yamaji, Daisuke Yamane, Mitsuko Hara, Keisuke Munekata, Kyoko Tsukiyama-Kohara, Tsunekazu Hishima, Soichi Kojima, Kiminori Kimura, Michinori Kohara

**Affiliations:** 1 Department of Microbiology and Cell Biology, Tokyo Metropolitan Institute of Medical Science, Setagaya-ku, Tokyo Japan; 2 Division of Hepatology, Tokyo Metropolitan Komagome Hospital, Bunkyo-ku, Tokyo Japan; 3 Department of Pathology, Tokyo Metropolitan Komagome Hospital, Bunkyo-ku, Tokyo Japan; 4 Micro-signaling Regulation Technology Unit, RIKEN Center for Life Science Technologies, Saitama, Japan; 5 Transboundary Animal Diseases Center, Joint Faculty of Veterinary Medicine, Kagoshima University, 1-21-24 Korimoto, Kagoshima, 890-0065 Japan

## Abstract

Chronic hepatitis C virus (HCV) infection is one of the major causes of serious liver diseases, including liver cirrhosis. There are no anti-fibrotic drugs with efficacy against liver cirrhosis. Wnt/β-catenin signaling has been implicated in the pathogenesis of a variety of tissue fibrosis. In the present study, we investigated the effects of a β-catenin/CBP (cyclic AMP response element binding protein) inhibitor on liver fibrosis. The anti-fibrotic activity of PRI-724, a selective inhibitor of β-catenin/CBP, was assessed in HCV GT1b transgenic mice at 18 months after HCV genome expression. PRI-724 was injected intraperitoneally or subcutaneously in these mice for 6 weeks. PRI-724 reduced liver fibrosis, which was indicated by silver stain, Sirius Red staining, and hepatic hydroxyproline levels, in HCV mice while attenuating αSMA induction. PRI-724 led to increased levels of matrix metalloproteinase (MMP)-8 mRNA in the liver, along with elevated levels of intrahepatic neutrophils and macrophages/monocytes. The induced intrahepatic neutrophils and macrophages/monocytes were identified as the source of MMP-8. In conclusion, PRI-724 ameliorated HCV-induced liver fibrosis in mice. We hypothesize that inhibition of hepatic stellate cells activation and induction of fibrolytic cells expressing MMP-8 contribute to the anti-fibrotic effects of PRI-724. PRI-724 is a drug candidate which possesses anti-fibrotic effect.

## Introduction

Liver fibrosis is a common feature of chronic hepatitis, and chronic liver injury leads to liver cirrhosis^1^. Regardless of the causes, liver fibrosis is characterized by an increase in the extracellular matrix (ECM) constituents that collectively form hepatic scars. Although liver fibrosis is becoming increasingly recognized as a major cause of morbidity and mortality in most chronic liver diseases, there are (to date) few—if any—treatment strategies available that specifically target the pathogenesis of fibrosis^2^. Following liver injury, hepatic stellate cells (HSCs) undergo an activation process and change their phenotype from quiescent retinoid-storing HSCs to collagen-producing and contractile myofibroblast-like cells. This transdifferentiation is characterized by reduced vitamin A content, increased cell proliferation, migration, and expression of α-smooth muscle actin (αSMA). Besides collagen production, the inhibition of ECM degradation is associated with progression of liver fibrosis^3^. ECM degradation is induced by the matrix metalloproteinase (MMP) family of enzymes. Overexpression of MMP-8, using a hepatitis B virus vector, ameliorates rat liver cirrhosis induced by thioacetamide^4^. Neutrophils are known to be producers of MMP-8; depletion of this cell type blocks collagen degradation in rat fibrotic liver^5^. In the resolving phase of liver fibrosis, increased MMP-8 activity and neutrophil accumulation are observed in the liver^6^. As with MMP-8, overexpression of MMP-9 in the mouse liver (using an adenovirus vector) reduces liver fibrosis after carbon tetrachloride (CCl_4_) treatment^7^. Additionally, hepatic macrophages are involved in the regression of hepatic fibrosis^8^; cells of this type also have been reported to produce MMPs^9^.

Wnt signaling affects developmental processes during embryogenesis and has an important role in tissue homeostasis in adults. Following Wnt activation, β-catenin translocates to the nucleus, where β-catenin binds to the T-cell factor/lymphoid enhancer-binding factor (TCF/LEF) to induce the transcription of Wnt target genes^10, 11^. Nuclear β-catenin/TCF then assembles a transcriptionally active complex by recruiting the transcriptional coactivators cyclic AMP response element binding protein (CREB) binding protein (CBP) or the closely related protein p300, as well as other components of the basal transcriptional machinery, to stimulate the transcription of target genes. The canonical Wnt signaling pathway has been implicated in the pathogenesis of a variety of tissue fibroses, including liver fibrosis^12–14^, and it has been shown that CBP/β-catenin antagonists are efficacious in a variety of injury models, including pulmonary and renal fibrosis^15, 16^. PRI-724 is a second-generation CBP/β-catenin-specific antagonist, a selective small-molecule inhibitor of β-catenin/CBP interaction, developed by PRISM Pharma Co., Ltd. (Kanagawa, Japan)^17^. PRI-724 treatment reduces liver fibrosis induced by CCl_4_ or bile duct ligation in mice^18^. In the present study, we examined whether PRI-724 has therapeutic potential for use in the treatment of liver fibrosis using an HCV transgenic mouse model. The results suggest that PRI-724 may be a candidate for a new anti-fibrotic drug.

## Results

### Intraperitoneal PRI-724 injection ameliorates hepatitis C virus-induced liver fibrosis

**Figure 1:**
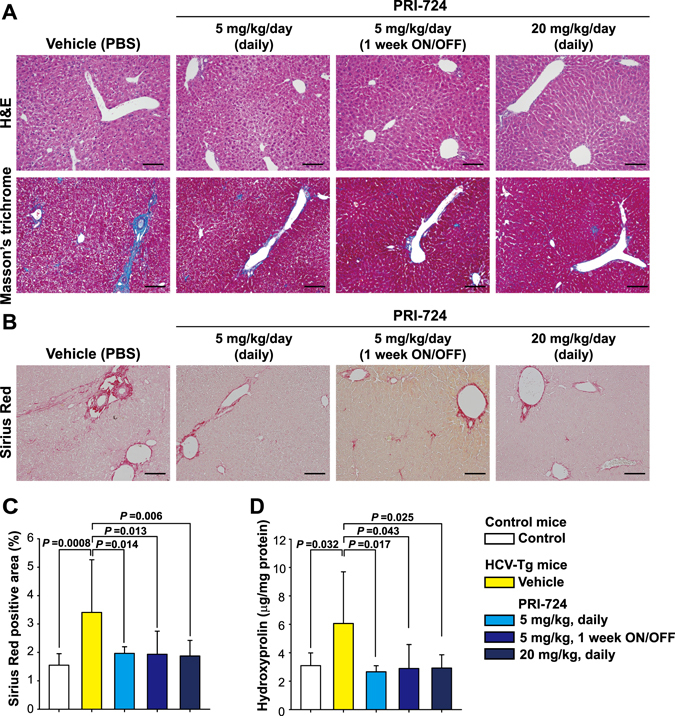
PRI-724, a selective inhibitor of β-catenin/CBP signaling, ameliorates hepatitis C virus-induced liver fibrosis. HCV transgenic mice were treated intraperitoneally with vehicle or PRI-724 once per day for 42 days at a dose of 5 or 20 mg/kg body weight or 1 week ON/OFF at a dose of 5 mg/kg body weight for 6 weeks (total of three cycles, each consisting of 1 week of once-daily dosing followed by 1 week without dosing) (Fig. S1A). Undosed non-trangenic mice were used as controls. (**A**) Liver damage and fibrosis were assessed by staining with hematoxylin and eosin (upper panels) or Masson’s trichrome (lower panels); representative micrographs are provided (scale bars = 200 μm). (**B**–**D**) Collagen deposition was assessed by Sirius Red staining and by measurement of hydroxyproline content. Sirius Red staining: Representative micrographs are provided in (**B**) (scale bars = 200 μm) and results are quantified in (**C**) (graph). Hydroxyproline content is quantified in (**D**) (graph). Plotted data are shown as the mean ± SD (control: n = 3; vehicle: n = 9; PRI-724 (5 mg/kg or 20 mg/kg, daily): n = 4; PRI-724 (5 mg/kg, 1 week ON/OFF): n = 3). Significance was assessed by Dunnett’s multiple comparison test as indicated; significant relationships are indicated by *P*-values.

To evaluate the anti-fibrotic activity of PRI-724 on liver fibrosis induced by HCV, HCV transgenic mice were injected intraperitoneally with PRI-724 once daily for 6 weeks at a dose of 5 or 20 mg/kg body weight or 1 week ON/OFF at a dose of 5 mg/kg body weight for 6 weeks (total of three cycles, each consisting of 1 week of once-daily dosing followed by 1 week without dosing) (Fig. S1A). In HCV transgenic mice, S100A4 expression, which is controlled by CBP/β-catenin, was increased; this induction was attenuated by the administration of PRI-724 (Fig. S2A). In vehicle-treated mice, abnormalities of liver plate arrangement and hepatocellular morphology with collagen deposition in the liver were observed, as shown by hematoxylin and eosin (H&E) and Masson’s trichrome staining (Fig. 1A) without ALT elevation (Fig. S3A). On the other hand, in PRI-724-treated mice, these abnormalities were attenuated and the area of collagen fibrils was reduced without reduction of HCV core protein expression (Fig. S3B). In particular, PRI-724 reduced collagen fibrils even in the case of 1 week ON/OFF treatment, suggesting that short-term discontinuation is tolerated for anti-fibrotic treatment by PRI-724. Sirius Red staining revealed that the increased area of collagen fibrils in the liver induced by HCV (3.4%; Sirius Red positive area/total area) was significantly attenuated (to 1.9 to 2.0%) by PRI-724 treatment (Fig. 1B and C). Similarly, the increase of hepatic hydroxyproline by HCV induction was attenuated following treatment with PRI-724 (Fig. 1D). These results clearly indicated that CBP/β-catenin signaling is activated in HCV-expressing liver, and that inhibition of CBP/β-catenin by PRI-724 is effective in counteracting HCV-induced liver fibrosis.

### PRI-724 inhibits HSC activation and promotes ECM degradation

**Figure 2:**
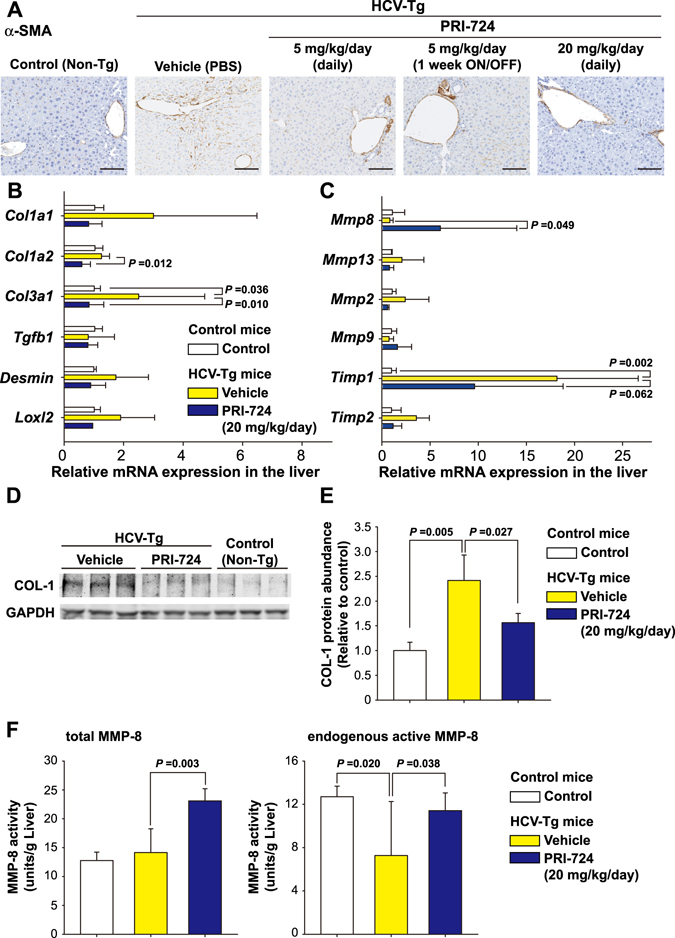
PRI-724 inhibits hepatic stellate cell (HSC) activation and induces matrix metalloproteinase-8 (MMP-8) expression. HCV transgenic mice were treated intraperitoneally with vehicle or PRI-724 once per day for 42 days at a dose of 5 or 20 mg/kg body weight or 1 week ON/OFF at a dose of 5 mg/kg body weight for 6 weeks (total of three cycles, each consisting of 1 week of once-daily dosing followed by 1 week without dosing) (Fig. 1A). Undosed non-trangenic mice were used as controls. (**A**) Expression of αSMA was examined by immunohistochemistry with anti-αSMA antibody; representative micrographs are provided (scale bars = 200 μm). (**B**,**C**) The liver expression of the indicated mRNA variants involved in collagen synthesis and maturation (**B**) or in collagen degradation (**C**) was determined by quantitative real-time RT-PCR in livers from non-transgenic control mice (white) or from HCV transgenic mice treated with vehicle (yellow) or with PRI-724 (20 mg/kg, once daily) (blue). Data are shown as the mean ± SD (control: n = 6; vehicle: n = 4; PRI-724 (20 mg/kg, daily): n = 4). (**D**,**E**) Expression of type I collagen (Col-1) in the livers of mice from the indicated groups was analyzed by western blotting. Densitometry was performed to define band density of (**D**), and values were normalized to GAPDH levels (**E**). Data are shown as the mean ± SD (control: n = 3; vehicle: n = 3; PRI-724 (20 mg/kg, daily): n = 3). Full length blots are included in Supplementary Information (Fig. S7). (**F**) Total MMP-8 (pro-MMP-8 plus active MMP-8) (left graph) and endogenous active MMP-8 (right graph) levels in the livers of mice from the indicated groups were assessed by measurement of collagenase activities against type II collagen. Data are shown as the mean ± SD (control: n = 4; vehicle: n = 4; PRI-724 (20 mg/kg, daily): n = 4). Significance was assessed by Dunnett’s multiple comparison test as indicated; significant relationships are indicated by *P*-values.

**Figure 3:**
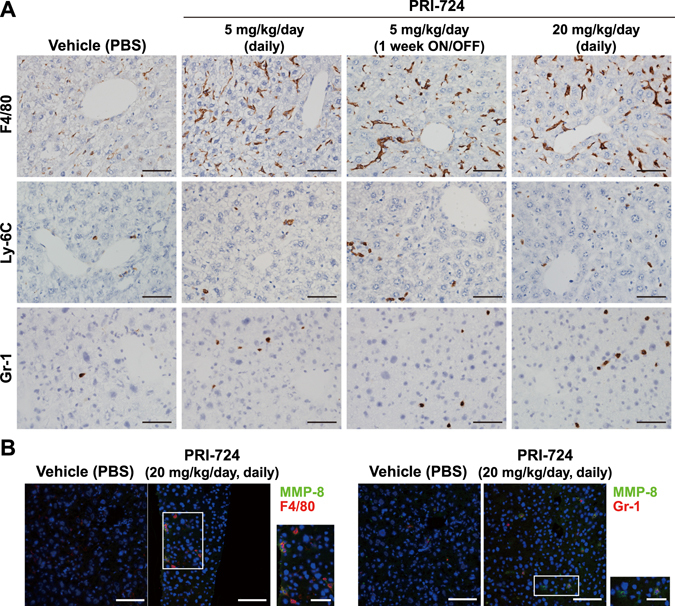
PRI-724 induces fibrolytic inflammatory cells expressing MMP-8. HCV transgenic mice were treated intraperitoneally with vehicle or PRI-724 once per day for 42 days at a dose of 5 or 20 mg/kg body weight or 1 week ON/OFF at a dose of 5 mg/kg body weight for 6 weeks (total of three cycles, each consisting of 1 week of once-daily dosing followed by 1 week without dosing) (Fig. 1A). Undosed non-trangenic mice were used as controls. (**A**) Expression of F4/80, Ly-6C, and Gr-1 was examined by immunohistochemistry with anti-F4/80, -Ly-6C, and -Gr-1 antibodies, respectively; representative micrographs are provided (scale bars = 200 μm). (**B**) Expression of MMP-8 (green) and cellular markers (red) F4/80 (left panels) and Gr-1 (right panels) was examined by immunofluorescent staining. Nuclei were counterstained with DAPI (blue). Representative images are provided (scale bars = 100 μm). MMP-8-producing cells in the PRI-724-treated group are boxed, and enlarged images are provided to the right of each original image (scale bars = 50 μm).

To investigate whether PRI-724 alters the activation status of HSCs in the liver, expression of αSMA was examined. αSMA expression was increased in HCV transgenic mice compared to control mice, and the induction of αSMA expression was attenuated by PRI-724 treatment (Fig. S2B). Similarly, immunohistochemistry revealed that the number of αSMA-expressing cells increased in HCV transgenic mice compared to control mice; this induction was attenuated by PRI-724 treatment (Fig. 2A). Moreover, the increase of collagen type 3 α1-encoding mRNA and type I collagen (Col-1) expression levels in the HCV transgenic mice also was attenuated by PRI-724 (Fig. 2B,D and E). These results suggested that HSC activation and collagen production in the transgenic mice is inhibited by PRI-724. In addition to the inhibitory effect of PRI-724 on HSC activation, expression of MMP-8-encoding mRNA was found to be enhanced 7.4-fold by PRI-724 treatment (Fig. 2C). Conversely, the HCV-related induction of an mRNA encoding TIMP-1 (an endogenous inhibitor of MMP-8) was attenuated by PRI-724 (Fig. 2C). Measurement of MMP-8 activities in the liver revealed that the total MMP-8 level (pro-MMP-8 plus active MMP-8) was increased by PRI-724 treatment and that the HCV-related reduction of endogenous active MMP-8 was attenuated by PRI-724 (Fig. 2F), suggesting that PRI-724 also is involved in the degradation of ECM by MMP-8. In contrast to the case with the *Mmp8* transcript, mRNA expression levels of *Mmp2*, *Mmp9*, and *Mmp13* were not affected by PRI-724 (Fig. 2C). However, total MMP-9 level was increased by PRI-724 treatment (Fig. S4), suggesting that MMP-9 may also contribute to remission of fibrosis by PRI-724. To identify the MMP-8-producing cells, immunostaining was performed. The numbers of F4/80-, Ly-6C-, and Gr-1-positive cells in HCV transgenic mice were elevated following treatment with PRI-724 (Fig. 3A). Moreover, MMP-8-positive cells also stained with both F4/80 and Gr-1 (Fig. 3B), suggesting that macrophages/monocytes and neutrophils are mobilized by PRI-724 and that these cells are producing MMP-8. Increased chemokine levels were not observed in PRI-724-treated animals (Table S1). The mechanism of macrophage and neutrophil recruitment in the livers of the PRI-724-treated mice will require further investigation.

### Intermittent administration of PRI-724 is sufficient for anti-fibrotic activity on hepatitis C virus-induced liver fibrosis

**Figure 4:**
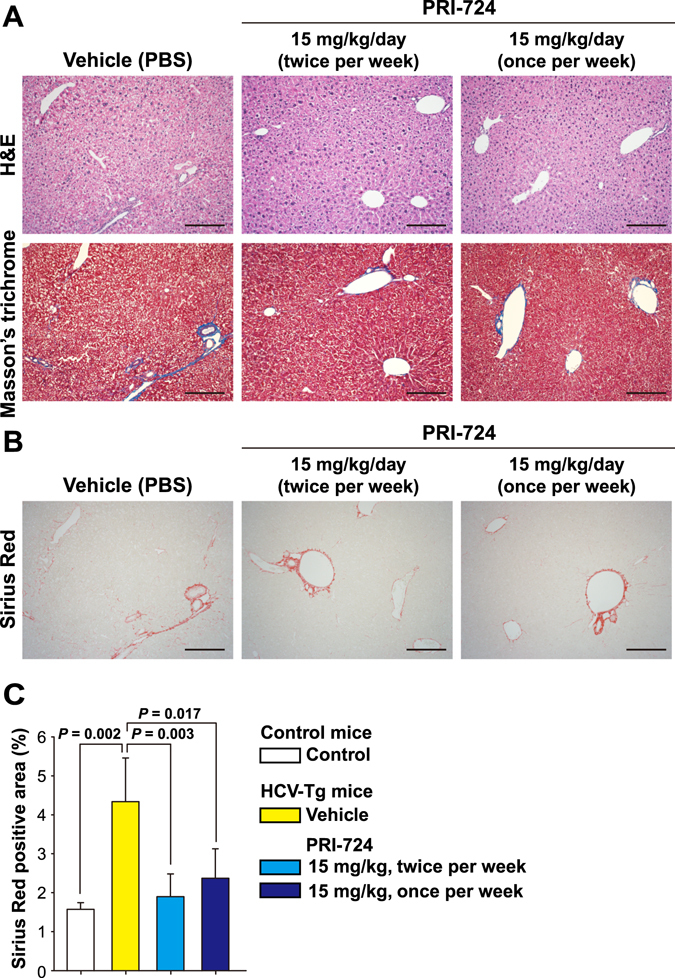
Intermittent administration of PRI-724 provides anti-fibrotic activity for HCV-induced liver fibrosis. HCV transgenic mice were treated with vehicle or PRI-724 (15 mg/kg body weight) administered intraperitoneally at a frequency of once or twice per week for 6 weeks (Fig. S1A). Undosed non-transgenic mice were used as controls. (**A**) Liver damage and fibrosis were assessed by staining with hematoxylin and eosin (upper panels) or Masson’s trichrome (lower panels); representative micrographs are provided (scale bars = 200 μm). (**B**,**C**) Collagen deposition was assessed by Sirius Red staining. Representative micrographs are provided in (**B**) (scale bars = 200 μm) and results are quantified in (**C**) (graph). Plotted data are shown as the mean ± SD (control: n = 3; vehicle: n = 3; PRI-724 (15 mg/kg, twice per week): n = 4; PRI-724 (15 mg/kg, once per week): n = 3). Significance was assessed by Dunnett’s multiple comparison test as indicated; significant relationships are indicated by *P*-values.

In clinical use, daily intraperitoneal injection is not feasible. Thus, the anti-fibrotic activity of intermittently administered PRI-724 was evaluated. HCV transgenic mice were intraperitoneally administered with PRI-724 (15 mg/kg body weight) at a frequency of once or twice per week (Fig. S1A). At six weeks after the initiation of treatment, the area of collagen fibrils in the liver was decreased in the PRI-724-treated group, as indicated by Masson’s trichrome staining (Fig. 4A). Sirius Red staining confirmed that both twice-weekly and once-weekly PRI-724 treatment provided a statistically significant attenuation in the HCV-induced increase in the area of collagen fibrils (Fig. 4B and C).

### Subcutaneous PRI-724 injection ameliorates hepatitis C virus-induced liver fibrosis

**Figure 5:**
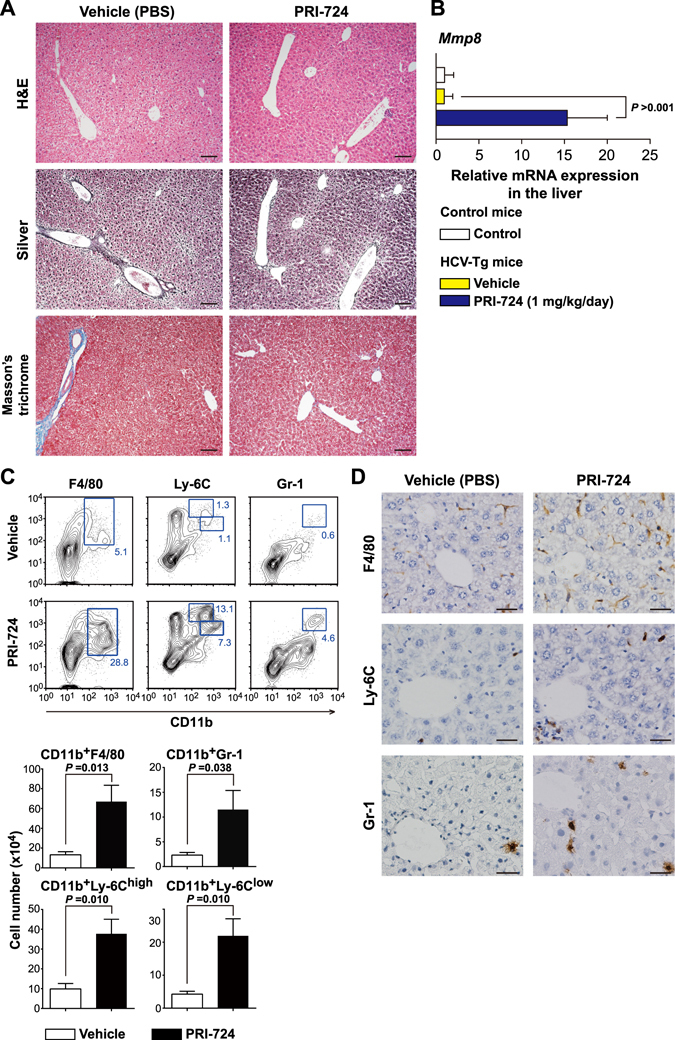
Subcutaneous continuous administration of PRI-724 is effective for treating fibrosis induced by HCV. HCV transgenic mice were treated with PRI-724 (1 mg/kg body weight) or vehicle (PBS) by continuous subcutaneous infusion for six weeks. Undosed non-transgenic mice were used as controls. (**A**) Liver damage and fibrosis were assessed by staining with hematoxylin and eosin (upper panels), silver stain (middle panels), and Masson’s trichrome (lower panels). Representative images are provided (scale bars = 100 μm). (**B**) The liver expression of *Mmp8* mRNA in non-transgenic control mice (white) and HCV transgenic mice treated with vehicle (yellow) or with PRI-724 (blue) was determined by quantitative real-time RT-PCR. (**C**) Representative scatter plots of each cell subset are shown in left panels. The absolute numbers of F4/80^+^CD11b^+^ (macrophages), Ly-6C^++^CD11b^+^ and Ly-6C^+^CD11b^+^ (monocytes), and Gr-1^+^CD11b^+^ cells (neutrophils) in intrahepatic leukocytes were determined by FACS (right panels). (**D**) Expression of F4/80, Ly-6C, and Gr-1 was examined by immunohistochemistry with anti-F4/80, -Ly-6C, and -Gr-1 antibodies. Representative images are provided (scale bars = 100 μm). Data are shown as the mean ± SD (control: n = 6; vehicle: n = 4; PRI-724 (1 mg/kg, daily): n = 4). Significance was assessed by Dunnett’s multiple comparison test as indicated; significant relationships are indicated by *P*-values.

As more suitable option for clinical use, we assessed the anti-fibrotic activity of PRI-724 using continuous subcutaneous infusion (Fig. S1B). As in the intraperitoneal injection model, subcutaneous administration of PRI-724 (1 mg/kg/day) effectively reduced the area of collagen fibrils in the liver, as assessed by silver staining and Masson’s trichrome staining (Fig. 5A). Administration of PRI-724 at 0.3 mg/kg/day also reduced the area of collagen fibrils in the liver, whereas the administration at 0.1 mg/kg/day did not show anti-fibrotic effect (Fig. S5), suggesting that PRI-724’s efficacy is dose dependent. As seen with intraperitoneal injection, subcutaneous dosing with PRI-724 yielded increases in the level of *Mmp8* mRNA expression and in the numbers of F4/80-, Ly-6C-, and Gr-1-positive cells (Fig. 5B,C and D). FACS analyses of intrahepatic leukocytes revealed that the numbers of both M1 (F4/80^+^CD11b^+^CD11c^+^CD206^−^ cells) and M2 macrophages (F4/80^+^CD11b^+^CD11c^−^CD206^+^ cells) were increased (Fig. S6). Further analysis will be needed to determine the identity and mechanism of the macrophages that contribute to the remission of fibrosis.

## Discussion

Cirrhosis is an increasing cause of morbidity and mortality in developed countries, being the 14^th^-most-common cause of death worldwide^1^. Organ fibrosis and cirrhosis are common end-stage diseases of the liver, resulting in life-threatening complications such as gastrointestinal bleeding and hepatocellular carcinoma^19^. The only available curative treatment for end-stage liver disease is transplantation of the liver, emphasizing the urgent need to identify new therapeutic strategies for the treatment of liver cirrhosis. Here, we demonstrated that liver fibrosis in HCV transgenic mice was significantly attenuated by treatment with PRI-724, a specific inhibitor of Wnt/β-catenin/CBP-driven transcription. These findings are consistent with a recent report that blockade of Wnt/β-catenin/CBP signaling by ICG-001 prevents bleomycin-induced pulmonary fibrosis in mice^16^. There is accumulating evidence of a role for Wnt/β-catenin signaling in the regulation of HSCs, a class of cells that have been implicated as key players in liver fibrosis^14, 20^. However, to our knowledge, the present study represents the first evidence that an inhibitor of the β-catenin/CBP interaction is capable of ameliorating liver fibrosis in the HCV transgenic mouse model.

The HCV transgenic mice used in the present study express multiple HCV proteins via the Cre/*loxP* switching system, resulting in the induction of persistent liver injury. As in humans, these mice subsequently develop steatosis, liver fibrosis, and hepatocellular carcinoma^21, 22^, suggesting that these mice serve as a good model of human HCV infectious liver diseases. Although there have been reports of experimental fibrosis models established by repeated administration of CCl_4_^23^ or by ligation of the bile duct^24^, these experimental models appear to be unsuitable for examining the effects of drugs on liver fibrosis, since these models do not involve the immunological responses, such as lymphocyte infiltration and interferon-γ synthesis, seen in human cases. In this context, we note that PRI-724 has relevant effects on the liver fibrosis observed in HCV transgenic mice, including the following: (1) PRI-724 regresses liver fibrosis in this model even after 18 months of continuous liver damage. (2) PRI-724 has anti-fibrotic effects even in older mice, which generally exhibit defects in tissue repair^25^. (3) PRI-724 does not induce liver damage when administered in healthy animals, and does not further damage HCV transgenic animals. Collectively, the above findings suggested that PRI-724 may be a promising therapy for the treatment of human liver fibrosis.

In the injured liver, HSC activation and fibrogenesis are mediated by complex cross-talk between damaged parenchymal and non-parenchymal cells. In our model, induction of αSMA expression by HCV was attenuated by PRI-724 treatment. Wnt signaling is stimulated in activated HSCs compared to that in quiescent cells, and the inhibition of Wnt signaling by the transduction of the adenoviral Wnt co-receptor antagonist Dickkopf-1 restores HSC quiescence and increases apoptosis in cultured HSCs^19^. Thus, HSCs might be the target cells of PRI-724 activity. To determine how PRI-724 exerts an anti-fibrotic function, we assessed the effect of this compound on expression of *Mmp8* mRNA, which encodes a matrix metalloproteinase that degrades ECM proteins in the liver. We observed significantly increased accumulation of the *Mmp8* transcript following PRI-724 treatment. Furthermore, immunohistochemical analysis revealed that macrophages and neutrophils in the liver produced MMP-8, suggesting that PRI-724 treatment induces the migration of these inflammatory cells into the liver. We postulate that the organ-localized activity of these cells facilitates regression of liver fibrosis. Recently, Ramachandran *et al*. reported that the CD11b^hi^ F4/80^int^ Ly-6C^lo^ subset of macrophages was most abundant in livers during maximal fibrosis resolution, and that this population represented the principal MMP-expressing subset^9^. Consistent with that report, our results also suggested that F4/80 macrophages and neutrophils are responsible for the resolution of liver fibrosis in HCV transgenic mice. However, the mechanism of inflammatory cell migration/recruitment to the liver in response to PRI-724 remains unclear.

Previously we reported the anti-fibrosis effects of PRI-724 in liver fibrosis induced by CCl_4_ or bile duct ligation in mice^18^. In this study, the anti-fibrosis effects of PRI-724 was confirmed in HCV-induced liver fibrosis, suggesting that PRI-724 is also effective in mouse model mimicking HCV infected patients. Moreover, our studies provide a proof of principle that the selective blockade of β-catenin/CBP signaling has potential as a novel therapeutic strategy for the treatment of liver fibrosis induced by various causes. In conclusion, PRI-724 ameliorated HCV-induced liver fibrosis in mice. Inhibition of HSC activation and induction of fibrolytic cells expressing certain matrix metalloproteinases might contribute to the anti-fibrotic effects of PRI-724. Thus, PRI-724 can be a candidate of anti-fibrotic drug.

## Methods

### Ethics Statement

This study was carried out in strict accordance with both the *Guidelines for Animal Experimentation* of the Japanese Association for Laboratory Animal Science and the recommendations in the *Guide for the Care and Use of Laboratory Animals* of the US National Institutes of Health. All protocols were approved by the ethics committee of Tokyo Metropolitan Institute of Medical Science.

### HCV transgenic mice

HCV GT1b transgenic mice (MxCre^+/−^/CN2-29^+/−^) were prepared as previously described^20^. HCV transgenic mice were injected intraperitoneally with 300 μg/mouse of polyinosinic acid-polycytidylic acid (poly(I)·poly(C)) (GE Healthcare, Buckinghamshire, UK) three times at 48-hour intervals to induce the expression of HCV proteins. Injection of poly(I)·poly(C) induces interferon production and the expression of CN2-29 gene products (HCV core, E1, E2, p7, and NS2) in hepatocytes, non-mesenchymal cells (mainly in Kupffer cells and lymphocytes), and spleen, but not in most other tissues; induction is mediated via the Cre/*loxP* switching system and mimics chronic HCV infection. The HCV core protein was expressed consistently in hepatocytes for at least 600 days, but not expressed consistently in Kupffer cells, lymphocytes and spleen as previously described^20^. HCV transgenic mice were used in the present study after long-term (18- to 20-month) HCV expression had led to the development of liver fibrosis. Age-matched controls (MxCre^−/−^/CN2-29^+/−^) were used and are designated below as non-transgenic control or control animals.

### PRI-724 treatment

PRI-724 (PRISM Pharma Co., Ltd.) was diluted in phosphate-buffered saline (PBS) and injected intraperitoneally once daily at a concentration of 5 or 20 mg/kg body weight for 6 weeks. For the 1 week ON/OFF experiment, the reagent (5 mg/kg/day) was administered intraperitoneally for 6 weeks during which animals were treated for a total of three cycles, each consisting of 1 week of once-daily dosing followed by 1 week without dosing. In a separate experiment, PRI-724 was administered intraperitoneally at 15 mg/kg body weight at a frequency of once or twice per week for 6 weeks. Alternatively, PRI-724 was dosed by subcutaneous infusion (1 mg/kg/day) for 6 weeks using implanted ALZET Mini-osmotic pumps MODEL 2006 (DURECT, Cupertino, CA). On the last day of the respective treatment, each animal was humanely killed and the liver was recovered at necropsy. Separate segments of each animal’s liver were fixed in formalin (for standard histology and immunohistochemistry); embedded in optimal cutting temperature (OCT) compound and frozen at −80 °C (for cryosectioning followed by immunohistochemistry); or flash-frozen in liquid nitrogen (for analysis of hydroxyproline content and assays of RNA and protein expression).

### Immunohistochemistry

Liver tissues were fixed in 10% neutral buffered formalin and embedded in paraffin, and tissue sections (4 μm in thickness) were prepared. The sections were stained with hematoxylin and eosin (H&E). Masson’s trichrome staining, silver staining, and Sirius Red staining (Picrosirius red staining kit, Polysciences, Inc., Warrington, PA) also were performed to visualize collagen fibrils. The ratios of Sirius Red positive/total area (%) from all observed areas were measured using a BZ-X700 (Keyence Japan, Osaka, Japan) hybrid cell counter system; approximately 100 fields were inspected per liver section. For immunostaining of paraffin-embedded sections, the liver sections were subjected to antigen retrieval using either microwaving (heating to 98 °C for 10 min in 0.01 M citrate buffer, pH 6.0; for anti-αSMA staining) or protease digestion (room temperature for 1 min in 0.04% proteinase K in 50 mM Tris-HCl, pH 7.4; for anti-F4/80 and anti-Ly-6C staining). Treated sections then were stained with anti-αSMA (Dako Japan, Tokyo, Japan), anti-F4/80 (BioLegend, San Diego, CA), or anti-Ly-6C (BioLegend) antibodies.

For Gr-1 staining, frozen OCT-embedded liver sections (4 μm in thickness) were fixed with a 50–50% mixture (v/v) of −20 °C acetone-methanol for 20 min. The fixed sections were treated with anti-Gr-1 antibody (eBioscience, San Diego, CA). Following detection by DAB solution, nuclei were counterstained with Mayer’s hematoxylin.

### Western blotting

Blotting of electrophoretically separated protein extracts was performed using anti-collagen type I (Rockland Immunochemicals Inc., Limerick, PA), anti-S100A4 (Abcam, Cambridge, UK), anti-αSMA (Abcam), and anti-glyceraldehyde-3-phosphate dehydrogenase (GAPDH) (WAKO, Osaka, Japan) antibodies. Band intensity was quantified by densitometry using Multi Gauge ver. 3.0 software (Fujifilm, Tokyo, Japan), and values were normalized to GAPDH levels.

### Measurement of hepatic hydroxyproline content

Approximately 40 mg of frozen liver tissue was homogenized in 500 μL of 2N NaOH for 10 min at 65 °C. A small aliquot of homogenate was used to quantify total protein by BCA assay. For the rest of the homogenate, an equal volume of 6N HCl was added, and samples were hydrolyzed for 20 min at 120 °C. Activated charcoal solution (10 mg/ml in 4N KOH) and 2.2M acetic acid-0.48M citric acid buffer (pH 6.5) were added, yielding a final pH of 7–8. After centrifugation at 15,000 rpm and room temperature for 10 minutes, chloramine-T solution (100 mM) was stirred into the supernatant for 25 min at room temperature. After the addition of 1 M Ehrlich’s solution (*p* dimethylaminobenzaldehyde), samples were incubated for 20 min at 65 °C. Absorbance was measured at 560 nm.

### Measurement of HCV core protein expression in the liver

HCV core protein levels in the liver from HCV transgenic mice were examined using an ELISA kit (Ortho-Clinical Diagnostics, Tokyo, Japan).

### Gene expression analysis

To measure the levels of liver mRNA, total RNA was extracted from the flash-frozen liver tissues (obtained from HCV transgenic mice or from age-matched non-transgenic control mice) by the acid guanidinium-phenol-chloroform method. cDNA, which was synthesized from total RNA from the frozen liver tissue using a High Capacity cDNA Reverse Transcription Kit (Applied Biosystems, Foster City, CA), was used for qPCR performed using TaqMan Gene Expression Assays (Applied Biosystems) and the CFX96 real-time PCR detection system (Bio-Rad Laboratories, Tokyo, Japan). Relative gene expression was quantified using the 2^−ΔΔCT^ method. 18 S rRNA was used as an internal control to normalize all data; the age-matched controls were used as the calibrator.

### Measurement of MMPs activity

Measurement of endogenous active MMP-8 and -9, or total MMP-8 and 9 activity was performed using a MMP-8 Activity Assay Kit (Life Laboratory Company, Yamagata, Japan) or a QuickZyme Mouse MMP-9 activity assay (QuickZyme, Lwiden, Netherlands) according to the manufacturer’s instructions. Liver homogenates were prepared from frozen liver tissues (obtained from HCV transgenic mice or age-matched non-transgenic control mice) with lysis buffer (50 mM Tris-HCl, pH 7.5, containing 0.1% Triton X-100, 0.2 M NaCl, and 5 mM CaCl_2_). Activity of endogenous active MMP-8 in the liver homogenate was measured as cleavage of FITC-labeled type II collagen. Measurement of total MMP-8 activity was done by the same method, instead using liver homogenates that had been treated with 1 mM *ρ*-aminophenyl mercuric acetate (APMA) for 6 hours at 37 °C.

### Immunofluorescence staining

For immunofluorescence analysis, frozen liver sections (4 μm in thickness) were fixed with 100% acetone at −20 °C for 20 min. Fixed sections were treated with blocking buffer (PBS supplemented with 1% bovine serum albumin and 2.5 mM EDTA) for 30 min at room temperature. MMP-8-producing cells were detected by double-staining with anti-MMP-8 (Abnova, Taipei, Taiwan) in combination with an antibody against a cell surface marker (either anti-F4/80 antibody or anti-Gr-1 antibody; both were obtained from BioLegend). After incubation with primary antibodies for 2 hours at room temperature, tissue sections were further incubated with secondary antibodies conjugated with AlexaFluor 488 (for MMP-8) or AlexaFluor 594 (for F4/80 and Gr-1) for 1 hour at room temperature. The nuclei were stained with DAPI (Molecular Probes; Invitrogen, Life Technologies, Carlsbad, CA), and the sections were encapsulated with SlowFade Gold Antifade Reagent (Molecular Probes). Stained tissues were viewed with a LSM780 laser-scanning confocal microscope (Carl Zeiss, Oberkochen, Germany).

### Isolation of intrahepatic leukocytes

To isolate intrahepatic leukocytes from HCV transgenic mice treated with PRI-724 or vehicle, single-cell suspensions were prepared from liver by PBS perfusion via the inferior vena cava and digestion in 10 mL of RPMI 1640 medium (Nissui Pharmaceutical Co., Ltd., Japan) containing 0.02% (w/v) collagenase IV (Invitrogen) and 0.002% (w/v) DNase I (Sigma-Aldrich) for 40 min at 37 °C. The digested cells were overlaid on Lympholyte M (Cedarlane, Burlington, Canada) according to manufacturer’s instructions. After density separation, isolated cells at the interface were recovered, washed, and counted, then used for fluorescence-activated cell sorting (FACS) analysis according to manufacturer’s instructions (BD Biosciences, San Jose, CA).

### FACS analysis

Intrahepatic leukocytes were incubated with anti-mouse CD16/32 antibody (BD Biosciences, San Jose, CA) for 10 min on ice to block binding to FcγRII/III. Then, cells were stained (for 20 min at 4 °C) with fluorescence-conjugated antibodies for cell surface markers. Fluorescence-conjugated monoclonal antibodies were obtained from BioLegend as follows: Pacific Blue-conjugated anti-mouse CD11b, allophycocyanin-Cy7-conjugated anti-mouse F4/80, FITC-conjugated anti-mouse Ly-6C, and phycoerythrin-conjugated anti-mouse Gr-1 antibody. Cells were fixed using the Cytofix/Cytoperm kit (BD Biosciences), then resuspended in 1 mL FACS buffer for analysis with the FACS Canto II system (BD Biosciences).

### Statistical analysis

Statistical analysis was performed with the JMP 12.2.0 software (SAS Institute Inc.; Cary, NC). Statistical differences between different groups were analyzed by One-way Analysis of Variance (ANOVA) on ranks with post-hoc Dunnett’s test (multiple comparisons versus a control). *P*-values of <0.05 were considered statistically significant.

## Electronic supplementary material

Supplementary Information
