# Supplementary material for: Selective inhibitor of Wnt/β-catenin/CBP signaling ameliorates hepatitis C virus-induced liver fibrosis in mouse model
Source: Sci Rep. 2017 Mar 23;7:325. doi: 10.1038/s41598-017-00282-w (PMC5427997; doi:10.1038/s41598-017-00282-w)
Supplement: Supplementary file 1 — Supplementary Information [file 41598_2017_282_MOESM1_ESM.doc]

**Supplementary information**

**Selective inhibitor of Wnt/β-catenin/CBP signaling ameliorates hepatitis C virus-induced liver fibrosis in mouse model**

Yuko Tokunaga, Yosuke Osawa, Takahiro Ohtsuki, Yukiko Hayashi, Kenzaburo Yamaji, Daisuke Yamane, Mitsuko Hara, Keisuke Munekata, Kyoko Tsukiyama-Kohara, Tsunekazu Hishima, Soichi Kojima, Kiminori Kimura, and Michinori Kohara

Supplementary information includes 4 Supplementary Figures and 1 Supplementary Table.

**Supplementary figures**

**Fig. S1. Schematic diagram illustrating experimental protocol used for PRI-724 treatment of HCV transgenic mice.**

**Fig. S2. Inhibition of CBP/β-catenin suppresses activation of HSCs.**

HCV transgenic mice were treated intraperitoneally with vehicle or PRI-724 (20 mg/kg/day) for 42 days. Protein extracts from the livers were subjected to SDS-PAGE, and immunoblotting was performed with anti-S100A4, anti-αSMA, and anti-GAPDH antibodies. Densitometry was performed to define band intensity and values (provided below the respective lanes) were normalized to GAPDH levels.

**A**

**
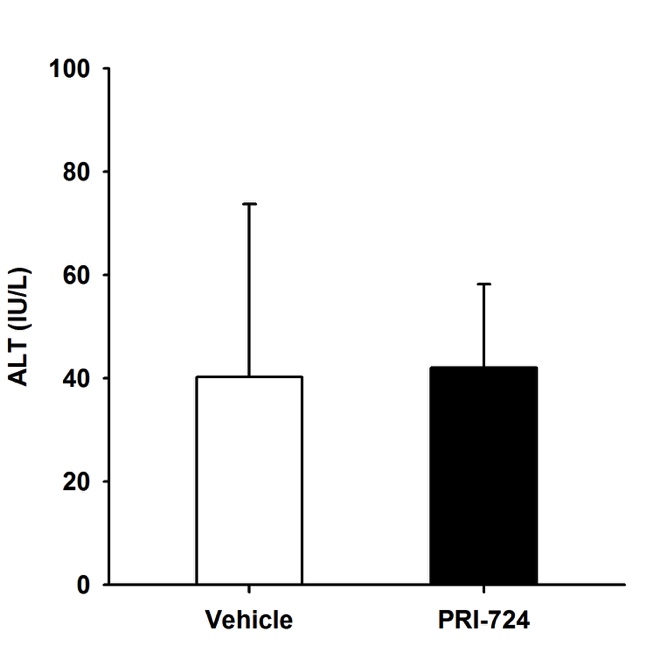
**

**B**


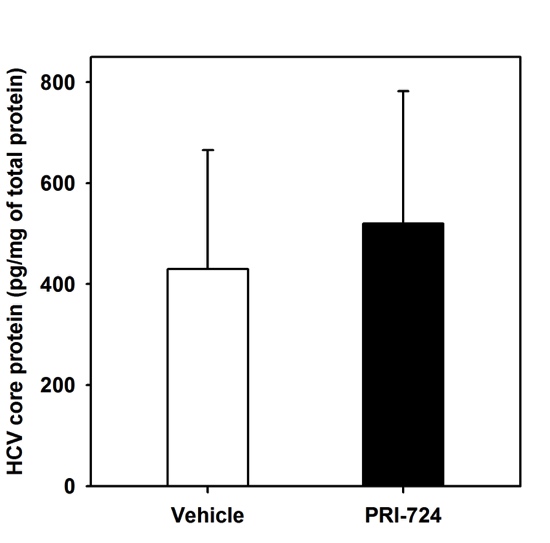


**Fig.S3. (A) Serum ALT levels in the HCV transgenic mice do not change during PRI-724 treatment.**

Serum ALT levels at six weeks after treatment of vehicle (white bar) or PRI-724 (black bar) were measured. Data are shown as the mean + SD (vehicle: n=9; PRI-724 (20 mg/kg, daily): n=4). Significance was assessed by Dunnett’s multiple comparison test as indicated; significant relationships are indicated by *P*-values.

**(B) PRI-724 treatment did not cause change in the expression of HCV core protein.**

HCV core protein levels in the liver from HCV transgenic mice treated with vehicle (white, n=3) or PRI-724 (black, n=4) for six weeks were measured using a commercial ELISA kit (Ortho-Clinical Diagnostics).


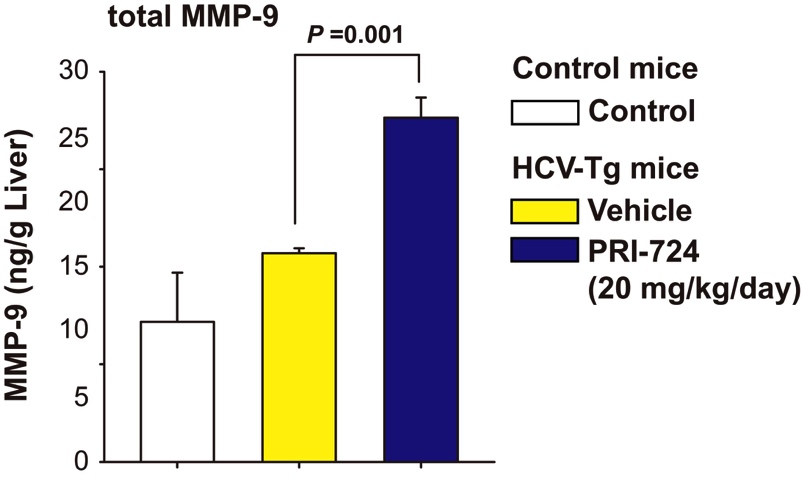


**Figure S4**. **MMP-9 expression levels in the liver are increased by PRI-724 treatment.**

Total MMP-9 levels in the liver from non-transgenic control mice (white) and HCV transgenic mice treated with vehicle (yellow) or PRI-724 (blue) for six weeks were measured by using QuickZyme Mouse MMP-9 activity assay (QuickZyme) after incubation with 0.25 mM *p*-aminophenyl mercuric acetate for 6 hours. However, the endogenous active MMP-9 could not detected by QuickZyme.

**Fig. S5. PRI-724 reduces collagen fibrils in the liver in a dose-dependent manner.**

PRI-724 (at 1.0, 0.3, or 0.1 mg/kg/day) or PBS (vehicle) was dosed by continuous subcutaneous infusion for six weeks using implanted osmotic pumps. Liver fibrosis was monitored by histology with silver stain (upper panels) and Masson’s trichrome staining (lower panels).

**Fig. S6. The numbers of M1 and M2 macrophage subsets are increased after PRI-724 treatment.**

HCV transgenic mice were treated with PRI-724 (1 mg/kg/day) or vehicle by continuous subcutaneous infusion for six weeks. Representative scatter plots of each cell subset at the end of treatment are shown in left panels.the Cell population (% of intrahepatic leukocytes) of F4/80^+^CD11b^+^CD11c^+^CD206^-^ cells (M1 macrophages) and F4/80^+^CD11b^+^CD11c^-^CD206^+^ cells (M2 macrophages) were determined by FACS (right panels). Statistical differences between different groups were analyzed by One-way Analysis of Variance (ANOVA) on ranks with post-hoc Dunnett’s test (multiple comparisons versus a control).

**Fig. S7.** **Expression of type I collagen (Col-1) in the livers of mice from the indicated groups was analyzed by western blotting in full size**.

Sample #1, 3, 4: HCV-Tg, Sample #5, 6, 8. Non-Tg #9, 10, 11.

**Supplementary table**

**Table S1. Relative cytokine expression in the liver of PRI-724- or vehicle-treated HCV transgenic mice compared to that of non-transgenic cont**rol mice.

| Cytokine | HCV transgenic mice | |
| --- | --- | --- |
|  | Vehicle | PRI-724 (20 mg/kg, daily) |
| IL-1α | 1.7±2.9 | 0.5±0.3 |
| IL-1β | 2.0±1.8 | 0.8±0.5 |
| IL-2 | 0.8±0.4 | 0.9±0.1 |
| IL-5 | 1.3±0.6 | 0.5±0.3 |
| IL-6 | 0.7±0.4 | 0.7±0.3 |
| IL-9 | 0.8±0.6 | 1.0±0.1 |
| IL-10 | 1.8±1.6 | 0.7±0.5 |
| IL-12 (p40) | 1.5±1.2 | 0.6±0.3 |
| IL-12 (p70) | 1.6±1.3 | 0.7±0.4 |
| IL-13 | 1.8±1.4 | 0.7±0.2 |
| IL-17 | 0.9±0.5 | 0.7±0.6 |
| Eotaxin | 1.4±1.3 | 0.6±0.4 |
| G-CSF | 10.0±18.7 | 0.7±0.7 |
| GM-CSF | 1.4±0.5 | 0.7±0.4 |
| IFN-γ | 1.4±0.9 | 0.8±0.1 |
| KC (CXCL1) | 1.7±1.6 | 1.4±1.5 |
| MCP-1 | 5.5±12.6 | 0.6±0.4 |
| MIP-1α | 5.9±9.4 | 0.8±0.4 |
| MIP-1β | 5.4±9.8 | 0.8±0.4 |
| RANTES (CCL5) | 1.6±1.1 | 1.3±0.9 |
| TNFα | 0.6±0.2 | 0.6±0.2 |
| IL-15 | 1.0±0.4 | 0.7±0.1 |
| IL-18 | 1.0±1.2 | 0.7±0.4 |
| bFGF | 0.9±0.4 | 0.6±0.3 |
| LIF | 2.1±2.7 | 0.8±0.3 |
| M-CSF | 1.3±0.8 | 0.7±0.4 |
| MIG (CXCL9) | 3.4±5.0 | 0.6±0.6 |
| MIP-2 (CXCL2) | 2.6±5.6 | 0.5±0.2 |
| PDGF-BB | 0.8±0.3 | 0.9±0.8 |
| VEGF | 1.7±3.1 | 0.3±0.1 |

Cytokines and chemokines in liver whole tissue lysates were measured by ready-made Bio-Plex mouse cytokine 23-Plex and 9-Plex Panel (Bio-Rad Laboratories) according to the manufacturer’s instructions. Relative expression levels in the liver of vehicle-treated (n=12) or PRI-724 (20 mg/kg/day, once daily)-treated (n=4) HCV transgenic mice compared to those of non-transgenic control mice (n=5) are shown.
